# Predictors of Postoperative ICU Admission in Patients With COVID-19-Associated Mucormycosis

**DOI:** 10.7759/cureus.29543

**Published:** 2022-09-24

**Authors:** Vibhor Gupta, Aman Yadav, Geetanjali T Chilkoti, Medha Mohta, Rajesh K Meena

**Affiliations:** 1 Anesthesiology and Critical Care, University College of Medical Sciences (University of Delhi) and Guru Tegh Bahadur Hospital, New Delhi, IND; 2 Anaesthesia, Banaras Hindu University, Varanasi, IND

**Keywords:** invasive ventilation, icu fatality, steroids, perioperative factors, icu, comorbidity, covid-19 associated mucormycosis

## Abstract

Background: Studies exploring factors predicting postoperative ICU requirement in patients with coronavirus disease 2019 (COVID-19)-associated mucormycosis (CAM) were not found in the literature. The aim was to evaluate the demographic profile, comorbidities, pattern of steroid received, airway assessment, and intraoperative hemodynamic perturbations associated with ICU requirement amongst patients scheduled for sinonasal debridement.

Methods: This is a retrospective cohort study. All CAM patients of ≥18 years were included. The patients’ characteristics, comorbidities, pattern of steroid received, airway assessment, intraoperative hemodynamic perturbations, and outcome data were retrieved.

Results: A total of 130 patients were included. Thirty got admitted to ICU, out of which 26 expired. Amongst the various comorbidities, diabetes was the most common (93.85%) and was associated with higher chances of ICU requirement. Of patients with a history of steroid intake, 71% had a significantly higher risk of ICU admission. Out of 30 patients admitted to ICU, 87% (n=26) received invasive ventilation, and the rest were admitted for observation only.

Conclusion: Middle age, uncontrolled diabetes, history of steroid intake, increased levels of serum creatinine with low potassium, and increased total leucocyte count are the independent risk factors predicting postoperative ICU admission amongst patients with CAM scheduled for sinonasal debridement.

## Introduction

Severe acute respiratory syndrome coronavirus 2 (SARS-CoV-2) causes coronavirus disease 2019 (COVID-19), which is associated with a wide range of bacterial and fungal infections that co-exist during or following the disease [1-5]. One of these infections is mucormycosis, which is angioinvasive in nature. A sharp surge in cases occurred during the COVID-19 pandemic. Specifically, the prevalence of COVID-19-associated mucormycosis (CAM) has varied from 0.005 to 1.7 per million population globally [6]. The prevalence of CAM in India is nearly 80 times the average in other developed countries [7]. Researchers have pointed to the ample presence of *Mucorales* in Indian communities, the large number of diabetic patients, and the neglect of regular health check-ups in the country as probable explanations for this outcome [8].

Anesthesiologists often encounter CAM patients when they are scheduled for neurosurgery, ophthalmological surgery, or oro-sinonasal debridement [9]. Patients scheduled for sinonasal debridement often have associated difficult airways in addition to the aforementioned comorbidities [10]. We conducted a literature search that retrieved no studies that have explored the various factors and anesthetic concerns relating to patients scheduled for surgery for the management of CAM.

In the present retrospective observational cohort study, we evaluated the demographic profiles, comorbidities, patterns of steroid and other treatments, airway assessments, and intraoperative hemodynamic perturbations and complications associated with postoperative ICU visits by patients scheduled for either elective or emergency sinonasal debridement for CAM during the second wave of COVID-19 pandemic (March-May, 2021) in India.

## Materials and methods

The participants included patients who were scheduled for sinonasal debridement in the ENT operation theater (OT). The duration of the study was from May 24 (i.e., the inception of the mucormycosis) through July 31, 2021. During this period, the University College of Medical Sciences and Guru Tegh Bahadur Hospital in New Delhi, India, was the designated facility for the management of CAM cases following the second wave of COVID-19 in the country. We undertook this retrospective cohort study following approval from the Institutional Ethics Committee For Human Research, University College of Medical Sciences, New Delhi, India, for conducting human research (IECHR-2021-50-16-R1). 

We included all the adult patients (those over 17 years old) diagnosed with CAM scheduled for sinonasal debridement surgery during the study period in our sample and excluded obstetric and pediatric patients. We retrieved the case files retrospectively from the medical records department and coded them so as to maintain anonymity. The data was extracted manually. The data covered the patients’ characteristics, laboratory and radiological investigations, and treatments, such as patterns of steroid use, preoperative airway assessments, anesthetic practices including intraoperative hemodynamic parameters, blood loss, and outcomes. The data was checked twice, and a third researcher adjudicated the differences in their interpretations.

The demographic profiles of the patients in our sample included age, gender, American Society of Anesthesiologists (ASA) physical status classification, duration of surgery, co-morbidities such as hypertension, chronic obstructive pulmonary disease (COPD), and diabetes mellitus, and the duration of the hospital stay prior to surgery (i.e., total days from hospital admission till the day of surgery). We also recorded baseline laboratory and radiological investigations before the surgery as well as the details regarding any previous use of oxygen therapy or steroids for the management of COVID-19. For those with a history of steroid use, we also recorded the dose, duration, and time since ceasing this treatment. We noted from the case files the patients’ complete airway assessments, such as mouth opening, Mallampati grade (MPG), thyromental distance in the preoperative period, and Cormack-Lehane (CL) grading at the time of intubation as well as the method for securing the airway, whether awake fiberoptic bronchoscopy, fiberoptic bronchoscopy following induction of anesthesia, or use of any airway adjunct such as a stylet, McCoy laryngoscope, bougie, video laryngoscope, or tracheostomy. We also recorded from the case files the results of routine blood examination, including complete blood count, total leucocyte count (TLC), coagulation profile, and serum biochemical test (including renal and liver function and electrolytes) of the patients.

We noted from the case file as well any hemodynamic perturbations (i.e., hypotension or bradycardia) during the intraoperative period. We defined hypotension and bradycardia as a drop in the systolic blood pressure of 20% and heart rate of 20%, respectively, below the baseline recorded on the operation theatre table before the induction of anesthesia. When necessary, hypotension was managed with IV fluids and vasopressors, and bradycardia with 0.6 mg of atropine IV. As per the records, the absence of tachycardia or hypertension indicated an adequate depth of anesthesia with optimum neuromuscular blockade, and analgesia was maintained throughout the surgery. For the patients who experienced hemodynamic perturbations, we noted any history of steroid treatment for COVID-19 and the time since the treatment had ceased.

We further recorded the other treatments that the patients had received retrospectively from their case files, including amphotericin, anticoagulants, and steroids. We noted as well the outcome, that is, whether the patients were sent to a ward or the ICU and, if so, any critical event or final outcome. The factors mandating postoperative ICU admission for elective/emergency postoperative ventilation or for observation included the presence of various comorbidities, significant intraoperative blood loss, intraoperative hemodynamic perturbations, and prolonged duration of surgery. In cases of morbidity or mortality, we noted from the case files the evident cause, such as acute respiratory distress syndrome (ARDS), heart failure, septic shock, coagulopathy, or acute kidney injury, and the number of days on a ventilator. We compared the various preoperative parameters, including demographic characteristics, comorbidities, preoperative investigations, airway assessment, and intraoperative parameters between the patients who required ICU and those who did not to identify the independent predictors of ICU admission among the patients scheduled for sinonasal debridement to treat CAM.

We analyzed the data with IBM SPSS Statistics for Windows, Version 20.0 (Released 2011; IBM Corp., Armonk, New York, United States). We present the descriptions of variables as means, medians (IQRs), or proportions depending on the nature of the data. Since we retrieved the data retrospectively, we omitted the rows that contained three or more missing variables from the analysis, and we replicated the rows with one or two missing variables with the mean, IQR, or mode, again depending on the nature of the data. We used multivariate forward logistic regression to explore the association of such factors as clinical characteristics and laboratory parameters with the risk of ICU requirement and mortality. We included all of the parameters in the regression model after removing multicollinearity and estimating the probability of entry at 0.05. We considered p-values of less than 0.05 to be significant.

## Results

A total of 160 patients were scheduled for various mucormycosis surgeries during the study period, sinonasal debridement being the most common procedure. Out of 160 patients, 130 were scheduled for sinonasal debridement and all had a history of COVID-19 and, hence, were labeled to have CAM. Of these 130 patients, only 10 patients had received COVID-19 vaccination (one dose only). Table 1 shows the demographic profile. CAM was seen in the age group of 40 to 60 years and the middle-aged group had significantly more chances of postoperative ICU admission with more preponderance in males. The mean duration of surgery was two hours.

**Table 1:** Association between demographic profile and ICU admission. * Mann-Whitney test, ^†^ Fisher's exact test, ^‡^ Chi-square test

Demographic characteristics	ICU not required	ICU required	Total	P value
Age (years) (Mean±SD)	46 (40-55.5)	60 (48.5-63.5)	48 (41-60)	0.0003^*^
Gender				
Female	46 (46%)	8 (27%)	54 (42%)	0.059^‡^
Male	54 (54%)	22 (73%)	76 (58%)	
Comorbidities				
Diabetes mellitus	92 (92%)	30 (100%)	122 (94%)	0.197^†^
Any other co-morbidities	63 (63%)	22 (73%)	85 (65%)	0.297^‡^
History of steroid intake				
No	36 (36%)	2 (7%)	38 (29%)	0.001^†^
Yes	64( 64%)	28 (93%)	92 (71%)	
Duration of steroids(days)	16(14-20)	18(14-20)	16(14-20)	0.135^*^
Last stopped steroid (days)	16(10-20)	16.5(11-20)	16(10-20)	0.891^*^
History of oxygen therapy during COVID-19 treatment				
No	74(74%)	18(60%)	92(71%)	0.139^‡^
Yes	26(26%)	12(40%)	38(29%)	
Patients on amphotericin	84(84%)	26(87%)	110(85%)	

Amongst the various comorbidities, diabetes was the most common (94%) in these patients with more risk of ICU requirement in diabetic patients as compared to any other comorbidity. Sixty-five percent of patients had other comorbidities like hypertension, COPD, chronic kidney disease, coronary arterial disease, or cerebrovascular accident.

Out of 130, 92 patients had a history of steroid intake, of which 93% (n=28) had a significantly higher risk of ICU admission (p-value < 0.005). The average mean duration of steroid intake was 14-20 days. Four patients out of 130 were found to be currently on steroid treatment. The mean time since the stoppage of steroids was found to be 10-20 days. None of the patients who had received steroids earlier were observed to have developed hemodynamic perturbations intraoperatively. Also, four patients who were currently on steroids at the time of surgery also did not report any hemodynamic disturbance. Only 29% of patients had received oxygen therapy during their management of COVID-19. Of the total patients in the study, 85% (n=110) were on amphotericin, out of which 87% (n=26) patients required ICU admission.

The preoperative laboratory investigations are shown in Table 2. Fasting blood sugar, blood urea, and serum creatinine were deranged in 43% (n=56), 68% (n=89) and 50% (n=65) of the patients, respectively. TLC was significantly deranged in 35% (n=46) of patients. However, hemoglobin, sodium, and potassium levels were within the normal range in most of the patients. Patients with deranged values of blood sugar, serum creatinine, and TLC had significantly higher chances of requiring ICU admission in the postoperative period.

**Table 2:** Association between investigations and ICU admission. * Mann-Whitney test, † Fisher's exact test, ‡ Chi-square test g/dl: gram per decilitre; mg/dl: milligram per decilitre; mEq/l: milliequivalents per litre

Investigations	ICU not required(n=100)	ICU required(n=30)	Total	P value
Hemoglobin (g/dL)				
Hemoglobin (< 10g per dl)	39 (39%)	14 (47%)	53 (41%)	0.454^‡^
Normal hemoglobin	61 (61%)	16 (53%)	77 (59%)	
Fasting blood sugar (mg/dL)				
Normal	66 (66%)	8 (27%)	74 (57%)	0.0001^‡^
Raised (>200 )	34 (34%)	22 (73 %)	56 (43 %)	
Urea (mg/dL)				
Normal	39 (39%)	2 (7%)	41 (32%)	0.0006^†^
Deranged (>40mg per dl)	61 (61%)	28 (93%)	89 (68%)	
Creatinine (mg/dL)				
Normal	63 (63%)	2 (7%)	65 (50%)	<.0001^†^
Deranged (>1.2mg per dl)	37 (37%)	28 (93%)	65 (50%)	
Sodium (mEq/L)				
Normal	59 (59%)	20 (67%)	79 (61%)	0.451^‡^
Deranged (<130 or>150meq per l)	41 (41%)	10 (33%)	51 (39%)	
Potassium (mEq/L)				
Normal	57 (57%)	10 (33%)	67 (52%)	0.023^‡^
Deranged (<3.5meq per l))	43 (43%)	20 (67%)	63 (48%)	
Total leucocyte count (per cubic mm)				
Normal	76 (76%)	8 (27%)	84 (65%)	<.0001^‡^
Deranged (>11000/mm^3^)	24 (24%)	22 (73%)	46 (35%)	

Airway assessment revealed that 11% (n=14) of patients had difficult mask ventilation. Also, it was noted that 26% of patients had difficult laryngoscopy (Cormack-Lehane grading >2b) and 35% (n=46) had difficult intubation with MPG >2. In Table 3, in context to the use of different airway devices, over 35% (n=46) of patients required either ambuscope (24%) or video laryngoscope (11%); whereas, in 65% (n=84) conventional direct laryngoscopy was used to secure airway.

**Table 3:** Association between airway assessment and ICU admission. ^†^ Fisher's exact test

Anesthesia technique to secure airway	ICU not required (n=100)	ICU required (n=30)	Total	P value
Ambuscope	27 (27%)	4 (13%)	31 (24%)	0.124^†^
Direct laryngoscopy	64 (64%)	20 (67%)	84 (65%)	
Video laryngoscopy	9 (9%)	6 (20%)	15 (11%)	
Total	100 (100%)	30 (100%)	130 (100%)	
Difficult mask ventilation	10	4	14 (11%)	
Difficult laryngoscopy	20	14	34 (26%)	
Difficult intubation	36	10	46 (35%)	

Intraoperative parameters like hemodynamic perturbations and blood loss were also noted. Only 8% (n=10) of patients showed hemodynamic perturbations. Table 4 shows a significant correlation between intraoperative blood loss to postoperative ICU requirement as all the 20 patients who had massive blood loss got admitted to the ICU in the postoperative period. It was also noted that out of a total of 30 patients admitted to ICU, 26 (87%) expired and only four recovered and were shifted back to the ward and later discharged. Out of 30 patients admitted to ICU, 87% (n=26) received invasive ventilation, and the rest were admitted for observation only.

**Table 4:** Association between intraoperative parameters and ICU admission. * Mann-Whitney test, ^†^ Fisher's exact test

Outcome	ICU not required (n=100)	ICU required (n=30)	Total	p-value
Final outcome				
Expired	0 (0%)	26 (87%)	26 (20%)	<.0001^†^
Recovered	100 (100%)	4 (13%)	104 (80%)	
Intraoperative blood loss				
No	100 (100%)	10 (33%)	110 (85%)	<.0001^†^
Yes	0 (0%)	20 (67%)	20 (15%)	
Intraoperative hemodynamic perturbations	7	3	10(8%)	
Total duration since hospital admission (days)	60 (35-80)	28 (19.25-32.25)	50 (28-80)	<.0001^*^

Table 5 shows the various causes of death amongst subjects. Sepsis (65%,n=17) was the most common cause of mortality in these patients followed by acute respiratory distress syndrome (ARDS) (23%,n=6). Further, acute kidney injury and heart failure were the causes in 8% and 4% of patients, respectively.

**Table 5:** Various causes of death amongst study subjects. ARDS: acute respiratory distress syndrome

Cause of death	Frequency	Percentage
Acute kidney injury	2	8%
ARDS	6	23%
Heart failure	1	4%
Sepsis	17	65%
Total	26	100%

Multivariate forward logistic regression of independent risk factors is shown in Table 6. It was observed that old age, history of steroid intake, uncontrolled sugar, serum creatinine, serum potassium levels, and TLC are independent risk factors predicting the risk of ICU admission in patients scheduled for elective surgery.

**Table 6:** Multivariate forward logistic regression to determine the risk factors for ICU admission after removing multicollinearity. mg/dl: milligram per decilitre; mEq/l: milliequivalents per litre; COVID-19: coronavirus disease 2019

Requirement of ICU	Beta coefficient	Standard error	p-value	Odds ratio	Odds ratio Lower bound (95%)	Odds ratio Upper bound (95%)
Age (years)	0.257	0.086	0.003	1.293	1.092	1.531
Total duration since hospital admission(days)	-0.171	0.061	0.005	0.843	0.748	0.950
Raised sugar (uncontrolled diabetes)	4.543	1.810	0.012	93.947	2.703	3264.998
History of steroid intake	13.431	4.852	0.006	6.81E+05	50.484	9.19E+09
History of oxygen intake during COVID-19 treatment	-3.854	1.596	0.016	0.021	0.001	0.484
Urea (mg/dL)						
Normal				1.000		
Deranged	-4.913	1.882	0.009	0.007	0.000	0.294
Creatinine (mg/dL)						
Normal				1.000		
Deranged	4.290	2.091	0.040	72.974	1.213	4391.598
Potassium (mEq/L)						
Normal				1.000		
Deranged	-5.455	2.274	0.016	0.004	0.000	0.368
Total leucocyte count (per mm^3^)						
Normal				1.000		
Deranged	3.849	1.531	0.012	46.947	2.336	943.442

Multivariate forward logistic regression to find out the risk factors of mortality following the removal of multicollinearity is shown in Table 7. It reveals that deranged TLC and significant blood loss were significantly associated with mortality in these patients.

**Table 7:** Multivariate forward logistic regression to find out the risk factors of mortality after removing multicollinearity.

Mortality	Beta coefficient	Standard error	P value	Odds ratio	Odds ratio Lower bound (95%)	Odds ratio Upper bound (95%)
Total leucocyte count (per mm^3^)						
Normal				1.000		
Deranged	2.098	0.899	0.020	8.149	1.398	47.498
Blood loss (>1Litre)	3.891	1.497	0.009	48.949	2.604	920.175

## Discussion

This retrospective cohort study, therefore, showed middle age, a history of steroid intake, elevated serum creatinine, blood sugar, and TLC levels to be independent factors predicting the risk of postoperative ICU admission among patients scheduled for surgery for the management of CAM. This fungal infection is angioinvasive in nature, and the organism responsible is common in the environment. Increasing reports of this disease have created alarm and drawn attention to the need for an effective treatment since it is associated with an extremely high mortality rate [11]. The COVID-19 pandemic has been associated with a surge in mucormycosis cases in India, especially after the second wave of COVID-19 [12,13]. Patients with rhino-orbital-cerebral mucormycosis commonly receive sinonasal debridement as well as orbital exoneration and decompression. During these surgical procedures, the anesthetic management of the patients has become challenging because they have associated co-morbidities and difficult airways and may also be receiving nephrotoxic drugs such as amphotericin. Therefore, it is necessary to mandate the evaluation of the various predictors of postoperative morbidity and mortality for COVID-19 patients associated with mucormycosis, an issue that no previous study has explored.

Regarding the patients’ demographic profiles, our study is consistent with previous research showing that CAM is more prevalent in male patients than female [9,14-17]. Our findings are also consistent with those of a collaborative study of 2,826 CAM patients showing that their mean age was 51.9 years (with a range of 12-88 years) and that most were male (71%; n=1,993) [11]. Likewise, a multicentric study observed that the patients with CAM tended to be middle-aged (mean age 56.9 years) and male (80.2%) than non-CAM patients [12].

The literature is replete with studies that identify diabetes mellitus and steroid intake as common and pertinent risk factors for the development of CAM. Singh et al., in a systemic review of 101 cases of CAM patients, identified diabetes mellitus (83.3%) and corticosteroid therapy (76.3%) as the most common risk factors for the disease and reported mortality in 30.7% of the cases [9]. Likewise, Sen et al. found, in a study of 2,826 CAM patients, 87% of patients had a history of steroid use and 78% had diabetes mellitus [11]. Similarly, John et al. reported that 94% of the CAM patients in their study had diabetes mellitus [14]. Other studies have also observed that the majority of CAM patients had diabetes mellitus and were receiving corticosteroid therapy [9,12,13]. Similarly, Ravani et al. conducted a retrospective study and identified uncontrolled diabetes mellitus (97.7%), COVID-19 infection (61.2%), and corticosteroid use (61.2%) as significant risk factors with a mortality rate of 9.78% [15]. Sharma et al. studied 23 CAM patients with a history of steroid use during their COVID-19 treatment, of whom 21 were diabetic; of these patients, 12 had uncontrolled blood sugar levels [16]. The non-diabetic patients in another retrospective comparative study showed a better survival rate than the diabetic patients (70% and 51%, respectively), and the mortality rate was 7.4 times higher in the mucormycosis patients with diabetes mellitus [18]. Consistent with the information available in the existing literature, we found diabetes mellitus and steroid use to be strong independent risk factors predicting ICU admission following sinonasal debridement for the treatment of CAM.

The use of steroids to manage COVID-19 appeared to be an important risk factor predisposing patients to mucormycosis. The literature pertaining to the use pattern of these medications, such as dose and duration, is scant. Our results indicate that patients with a history of extensive steroid intake tend to have a relatively higher risk of ICU admission after surgery as compared to those without such a history. In a collaborative study, Sen et al. observed that cumulative doses of greater than 600 mg of prednisone and 2-7 g of methylprednisolone have been found to predispose immunocompromised patients to mucormycosis [11]. Hoang et al. reported observing this effect with short courses of corticosteroids based on the case of a 66-year-old man with well-controlled type 2 diabetes mellitus (hemoglobin A1c of 6.4) who received a cumulative dose of over 600 mg of prednisolone over two weeks after being diagnosed with influenza A and contracted life-threatening pulmonary mucormycosis [19]. For the present retrospective study, we were unable to retrieve the type, dose, or duration of steroid use for the management of COVID-19 because we lacked access to the case files of those who were hospitalized for the management of COVID-19. Since many of the patients were undergoing isolation and treatment at home, the details of their steroid use were not uniformly available.

Because of the involvement of the eyes and sinuses in the infection, these patients may endure difficult mask ventilation and intubation. In the only study of this issue of which we are aware, Karaaslan et al. observed that three of 12 patients experienced difficult intubation because of fungal debris [10]. Similarly, more than 35% of the patients in our study required either a flexible fiberoptic bronchoscope or a video laryngoscope to secure their airways, potentially because of epiglottitis and fungal debris.

Moreover, most of the patients in our study had deranged blood urea, serum creatinine, and potassium levels, probably as a result of amphotericin B therapy, and these patients had a higher risk of postoperative ICU admission and mortality as compared to those without these characteristics. Various studies have shown that amphotericin B has significant renal toxicity at high doses [20-22].

The main cause of mortality among the patients in our study was sepsis followed by ARDS. Hong et al. also found that disseminated infection leading to sepsis correlated with an increased risk of death in mucormycosis patients [23]. Other studies have shown that other underlying conditions predispose individuals to the infection, including trauma, burns, intravenous drug use, use of broad-spectrum antibiotics, increases in iron or ferritin in the system, malnutrition, and use of voriconazole [24-26]. Additional studies are needed to explore and identify other various risk factors.

The present study has some limitations. To begin with, a few of the laboratory tests, such as those for serum iron and serum ferritin, that have been found to predict infections were not conducted in all cases and hence, were not considered risk parameters. Also, we followed the patients only during the in-hospital course of treatment and had no access to the follow-up details of the survivors. Lastly, the generalizability of our findings may be limited by the sample size. Of note, more studies are needed to evaluate these factors for management.

## Conclusions

Upon our literature search, we could not retrieve any study exploring various factors predicting postoperative ICU requirement in patients scheduled for sinonasal debridement for CAM. Our study highlighted that middle age, uncontrolled diabetes, history of corticosteroid usage, increased serum creatinine, low serum potassium, and deranged TLC are independent risk factors predicting the postoperative ICU admission amongst patients of CAM scheduled for sinonasal debridement. There were certain limitations of this study such as a few laboratory tests like serum iron and serum ferritin were not conducted in all patients and hence were not included as risk parameters. Also, the study followed the patients during the in-hospital course only and, therefore, the follow-up details of the survivors could not be commented on. Lastly, the interpretation of our findings might be limited by the sample size. Hence, more studies are needed to evaluate these factors for management.
